# Increasing Fruit Weight by Editing a *Cis*-Regulatory Element in Tomato *KLUH* Promoter Using CRISPR/Cas9

**DOI:** 10.3389/fpls.2022.879642

**Published:** 2022-04-11

**Authors:** Qiang Li, Qian Feng, Ashley Snouffer, Biyao Zhang, Gustavo Rubén Rodríguez, Esther van der Knaap

**Affiliations:** ^1^College of Horticulture, Hebei Agricultural University, State Key Laboratory of North China Crop Improvement and Regulation, Key Laboratory of Vegetable Germplasm Innovation and Utilization of Hebei, Collaborative Innovation Center of Vegetable Industry in Hebei, Baoding, China; ^2^Center for Applied Genetic Technologies, University of Georgia, Athens, GA, United States; ^3^Instituto de Investigaciones en Ciencias Agrarias de Rosario (IICAR-CONICET-UNR), Cátedra de Genética, Facultad de Ciencias Agrarias UNR, Santa Fe, Argentina; ^4^Institute of Plant Breeding, Genetics and Genomics, University of Georgia, Athens, GA, United States; ^5^Department of Horticulture, University of Georgia, Athens, GA, United States

**Keywords:** tomato, *KLUH*, fruit weight, CRISPR/Cas, promoter, *cis*-regulatory element

## Abstract

CRISPR/Cas-mediated genome editing is a powerful approach to accelerate yield enhancement to feed growing populations. Most applications focus on “negative regulators” by targeting coding regions and promoters to create nulls or weak loss-of-function alleles. However, many agriculturally important traits are conferred by gain-of-function alleles. Therefore, creating gain-of-function alleles for “positive regulators” by CRISPR will be of great value for crop improvement. CYP78A family members are the positive regulators of organ weight and size in crops. In this study, we engineered allelic variation by editing tomato *KLUH* promoter around a single-nucleotide polymorphism (SNP) that is highly associated with fruit weight. The SNP was located in a conserved putative *cis*-regulatory element (CRE) as detected by the homology-based prediction and the Assay for Transposase-Accessible Chromatin using sequencing (ATAC-seq). Twenty-one mutant alleles with various insertion and deletion sizes were generated in the LA1589 background. Five mutant alleles (*m2_+4*bp*_*, *m3_+1*bp*_*, *m5_–1*bp*_*, *m13_–8*bp*_*, and *m14_–9*bp*_*) showed a consistent increase in fruit weight and a significant decrease in the proportion of small fruits in all experimental evaluations. Notably, *m2_+4*bp*_* and *m3_+1*bp*_* homozygote significantly increase fruit weight by 10.7–15.7 and 8.7–16.3%, respectively. Further analysis of fruit weight based on fruit position on the inflorescence indicated that the five beneficial alleles increase the weight of all fruits along inflorescence. We also found that allele types and transcriptional changes of *SlKLUH* were poor predictors of the changes in fruit weight. This study not only provides a way of identifying conserved CRE but also highlights enormous potential for CRISPR/Cas-mediated *cis*-engineering of CYP78A members in yield improvement.

## Introduction

As populations grow and the climate changes, demands for increased crop productivity continue across the world (Tilman et al., 2011; Ray et al., 2013). However, the rate of crop yield increase driven by conventional breeding technologies is not on pace to meet the increasing demands for food (Godfray et al., 2010; Ray et al., 2013; Long et al., 2015; Gao, 2018; Willmann, 2018; Hickey et al., 2019; Wolter et al., 2019). Yield and produce size are complex quantitative traits controlled by multiple genes. Despite enormous efforts made in the identification of yield-related genes in various crops, the implementation of these alleles in breeding further yield improvements is limited due to the low levels of genetic diversity stemming from long-term domestication and intensive selection (Zuo and Li, 2014; Kumar et al., 2017). Furthermore, yield and fruit size are regulated by numerous quantitative trait loci (QTLs) with subtle effects, and the identification and introgression of natural mutations are time-consuming and laborious (Doebley et al., 2006; Shi and Lai, 2015; Birchler, 2017). Therefore, the development of innovative technologies toward yield increases is essential to expand phenotypic diversity and accelerate yield enhancement to feed a growing population.

The CRISPR/Cas technologies have been successfully applied for crop improvement (Rodriguez-Leal et al., 2017; Gao, 2018; Chen et al., 2019; Hua et al., 2019; Mao et al., 2019; Zhang et al., 2019; Zhu et al., 2020). However, CRISPR/Cas-mediated genome editing has mainly focused on coding regions to produce loss-of-function mutants (Pandiarajan and Grover, 2018; Wolter and Puchta, 2018; Korotkova et al., 2019; Li Q. et al., 2020). While this application widely favors reverse genetic approaches for many domestication traits (Li et al., 2018; Zsogon et al., 2018), loss-of-function mutations often result in developmental defects that would hamper their applications in crop improvement (Xu et al., 2015; Swinnen et al., 2016; Morineau et al., 2017; Li C. et al., 2020). On the other hand, engineering *cis*-regulatory motifs (*cis*-engineering) within non-coding regions could result in fine-tuning gene expression and generate phenotypic diversity with less pleiotropic or deleterious effects than loss-of-function alleles (Swinnen et al., 2016; Rodriguez-Leal et al., 2017; Pandiarajan and Grover, 2018; Wolter and Puchta, 2018; Huang et al., 2020; Li C. et al., 2020; Liu et al., 2021).

The downregulation of gene expression as the result of the editing of *cis*-regulatory regions in genes which are negative regulators of a desirable trait has been successful in crop improvement (Duan et al., 2016; Jia et al., 2016; Holme et al., 2017; Peng et al., 2017; Rodriguez-Leal et al., 2017; Hummel et al., 2018; Li et al., 2018; Jia et al., 2019; Korotkova et al., 2019; Oliva et al., 2019; Huang et al., 2020; Li C. et al., 2020; Liu et al., 2021). However, many agriculturally important traits are conferred by dominant gain-of-function mutations (Korotkova et al., 2017; Li et al., 2017; Korotkova et al., 2019). Therefore, the generation of gain-of-function alleles in the promoters of positive regulators of traits could lead to the modulation of gene expression levels and tissue or temporal-specific expression patterns that would have great value for crop improvement.

CYP78A family members are recognized as positive regulators of organ weight and size in many crops, such as tomato (Zhang, 2012; Chakrabarti et al., 2013; Li et al., 2021), rice (Nagasawa et al., 2013; Yang et al., 2013; Maeda et al., 2019), wheat (Ma et al., 2015a,b), maize (Sun et al., 2017), soybean (Wang et al., 2015; Zhao et al., 2016), pepper (Chakrabarti et al., 2013), *Jatropha curcas* (Tian et al., 2016), and sweet cherry (Qi et al., 2017). Tomato *KLUH* (*SlKLUH*) underlies the fruit weight locus *fw3.2*. *SlKLUH* copy number is positively associated with fruit weight whereas knockout or knockdown *SlKLUH* often results in smaller fruits as well as other growth defects, including tiny inflorescences and infertile flowers (Chakrabarti et al., 2013; Alonge et al., 2020).

Previously, we identified a potential regulatory SNP, named M9 SNP, in the promoter of *SlKLUH* which is highly associated with fruit weight. Thus, the M9 SNP was proposed to be the causative variant of the *fw3.2* locus (Chakrabarti et al., 2013). However, a recent study demonstrated that an ∼50-kbp tandem duplication, rather than the M9 SNP, that includes *SlKLUH* underlies *fw3.2*, giving rise to 2- to 3-fold higher expression of *SlKLUH* and larger fruits. In this study, we found that the four tandem repeats are conserved motifs in the promoter of *SlKLUH* orthologs detected by homology-based prediction and Assay for Transposase-Accessible Chromatin using sequencing (ATAC-seq). The putative conserved motif was edited by CRISPR/Cas9 with a single guide RNA (gRNA) which included the M9 SNP to generate a total of 21 alleles. From these, we produced a series of homozygous transgene-free mutants showing a range of fruit weight variations. Among them, five mutant alleles (*m2_+4*bp*_*, *m3_+1*bp*_*, *m5_–1*bp*_*, *m13_–8*bp*_*, and *m14_–9*bp*_*) confer increased fruit weight with subtle effects in tomato in three experimental evaluations. Our data indicate that within tomato, *cis*-engineering of “positive regulators” using CRISPR/Cas9 has the potential for the improvement of quantitative traits. Furthermore, the application of *cis*-engineering of “positive regulators” to generate beneficial variants and the alleles generated in the conserved motifs in the promoter of *SlKLUH* identified are likely applicable to diverse crops.

## Materials and Methods

### Plant Materials and Growth Conditions

LA1589 carries a single copy of *fw3.2* and a wild-type (*wt*) allele of the M9 SNP. Seeds were sown directly in the soil in 72-count 6 pack trays and grown in a growth chamber under 16-h light/8-h dark photoperiod for 5–6 weeks. The 5- to 6-week-old seedlings were transplanted into 2.45 L pots and were cultivated in a greenhouse under 16-h light/8-h dark photoperiod in Athens, GA, United States. All the plants of each experimental evaluation were randomly arranged in the greenhouse.

### Guide RNA Design, CRISPR/Cas9 Construct, and Plant Transformation

The gRNA targeting the M9 SNP was designed using the CRISPR-P tool^1^ (Lei et al., 2014). The CRISPR/Cas9 construct was assembled using the Golden Gate cloning method as previously described (Wu et al., 2018). Electroporation was used to introduce the final binary vector into *Agrobacterium tumefaciens* strain LBA4404, which was kindly provided by Dr. Joyce Van Eck, Cornell University. The LBA4404 harboring the binary vector was used for the transformations of LA1589. The genetic transformations of LA1589 were performed as described (Gupta and Van Eck, 2016) at Wayne Parrott’s Laboratory, University of Georgia.

### Genotyping Edited Plants and Recovery of Homozygous Progenies

For genotyping of T_0_ transgenic plants, genomic DNA was extracted from leaves and was used for genotyping by PCR for the presence of the Cas9 using two specific primer pairs (14EP426/14EP427; 14EP438/14EP439) on the Cas9 coding region. The target region was amplified by PCR from the genomic DNA using specific primers flanking the gRNA target sites (17EP42/17EP47). The PCR products were purified and directly sequenced by Sanger sequencing. The mutations in T_0_ generation were analyzed by decoding sequencing chromatograms (Ma et al., 2015c).

T_0_ transgenic plants were backcrossed to LA1589, and the seeds of the F_1_ generation were extracted and sown in 288-well plastic flats. Each progeny was genotyped by PCR using primer flanking of the target region (17EP218/EP2460), and PCR products were resolved on 3.5% (w/v) agarose gel. The progenies harboring small indels that cannot be easily identified by PCR assay were further genotyped by Derived Cleaved Amplified Polymorphic Sequences (dCAPS)-*Bsr*BI, dCAPS-*Aci*I, and dCAPS-*Hae*III. For Cas9-free F_1_ plants, they were self-pollinated for the generation of homozygous mutants in the F_2_ generation. For the F_1_ plants carrying the Cas9 transgene, they were backcrossed with LA1589, and the Cas9-free homozygotes were obtained in the F_3_ generation. All the mutant alleles from the selected plants in F_1_, F_2_, and F_3_ generations were confirmed by Sanger sequencing, and the presence of Cas9 was determined by two specific primers in its coding region (Supplementary Table 4).

### Phenotyping

The phenotyping was performed on homozygous mutants with three experimental evaluations during 2018 and 2019, each with at least three plants per genotype (Supplementary Table 3). Three stems were kept and trained on a bamboo stick, respectively. We kept six inflorescences per plant with eight fruits on each inflorescence. All the flowers were pollinated by hand. We numbered the fruit position from 1 to 8 according to the proximal to distal positions on each inflorescence, and the inflorescences were numbered on each plant from 1 to 6 according to the harvesting time. The fruit weight was measured individually using a precision balance (VWR 64B). For fruit weight distribution analysis, fruits were grouped into four categories based on the quartiles of the fruit weight from all genotypes in each experimental evaluation.

### RNA Extraction and Quantitative Real-Time PCR

Since *SlKLUH* showed very low expression in developing fruits and very high expression in young flower buds in LA1589 (Chakrabarti et al., 2013), young flower buds at 9–13 days post initiation (dpi) (Supplementary Figure 7) were collected from at least three inflorescences per plant. Total RNA from meristems was then extracted using the TRIzol^®^ Reagent (Thermo Fisher, United States). Total RNA was used for cDNA synthesis with a High-Capacity cDNA Reverse Transcription Kit (Thermo Fisher, United States). Quantitative real-time PCR (qRT-PCR) was performed with gene-specific primers using the SsoAdvanced™ Universal SYBR^®^ Green Supermix (Bio-Rad, United States) reaction system on the CFX96 Real-Time system (Bio-Rad, United States), following manufacturer’s instructions. Clathrin adaptor complexes medium subunit (CAC) gene was used as an internal control (González-Aguilera et al., 2016; Supplementary Table 4).

### Statistical Analysis

Statistical analysis was performed in R. The information of the statistical test is given in the respective figures.

## Results

### Generation of Novel *Cis*-Regulatory Alleles by CRISPR/Cas9 in LA1589

**FIGURE 1:**
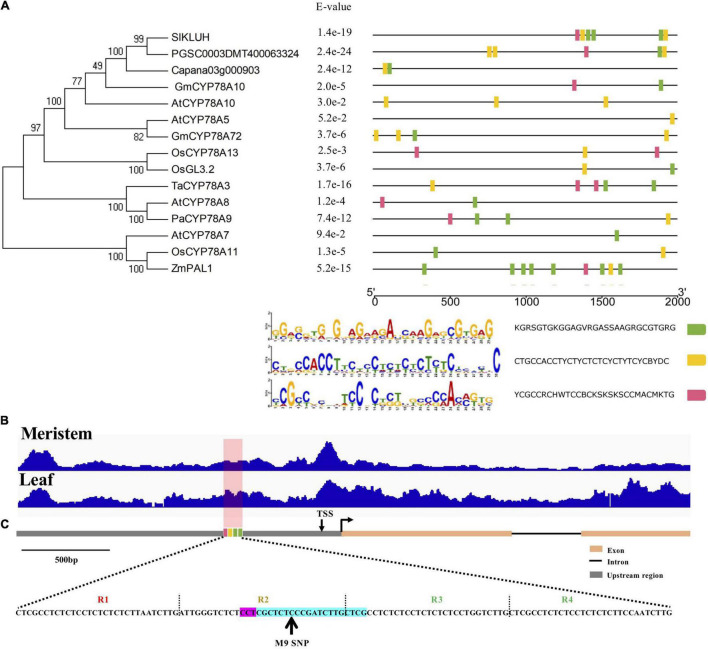
Identification of putative *cis*-regulatory element in *SlKLUH* promoter using homology-based prediction and ATAC-seq. **(A)** Conserved motif analysis in the promoters of CYP78A members. The unrooted phylogenetic tree was depicted by the MEGA program with the neighbor-joining (NJ) method using full protein sequences of 15 CYP78As from tomato (*Sl*), pepper (*Capana03g000903*), potato (*PGSC0003DMT400063324*), *Arabidopsis* (*At*), rice (*Os*), wheat (*Ta*), soybean (*Gm*), maize (*Zm*), and sweet cherry (*Pa*). To identify the conserved motifs, the 2-kb upstream region of the start codon of the *KLUH*s was analyzed in MEME with the following parameters: “nmotifs 3, minw 6, maxw 30”; **(B)** ATAC-seq indicated that the target region was located in open chromatin. The data were visualized by the Integrative Genomics Viewer. The peaks at the target region were indicated by red shading; **(C)** A schematic map of the gRNA-targeted site. The protospacer adjacent motif (PAM) site and gRNA were highlighted in pink and cyan, respectively. The four tandem repeats in promoter were indicated by R1, R2, R3, and R4. TSS, transcription start site.

The M9 SNP is located within the second repeat of four 30-bp tandem repeats (Supplementary Figure 1). Homology-based prediction using Multiple Em for Motif Elicitation (MEME) indicated that the four tandem repeats are conserved motifs in orthologous *KLUH* promoters (Figure 1A), suggesting that the motifs might be the important regulatory elements of the *CYP78A* genes. Since active gene regulatory elements are associated with open chromatin (Zhu et al., 2015; Lu et al., 2019; Yan et al., 2020), ATAC-seq was utilized in tomato meristem and leaf tissues (Hendelman et al., 2021). Significant peaks covering the repeats were detected in both tissues, indicating that this region might be an important regulatory region of *SlKLUH* (Figure 1B). Many genetic changes underlying traits of economic importance reside in *cis*-regulatory elements (CREs) and *cis*-engineering mediated by CRISPR/Cas for crop improvement can be utilized to expedite the modification of these CREs (Li Q. et al., 2020; Hendelman et al., 2021). Therefore, we hypothesized that engineering fruit weight variation could be implemented by editing the presumptive CRE of *SlKLUH*.

We tested the hypothesis by targeting the CRE using CRISPR/Cas9 with a single gRNA in the wild relative of the cultivated tomato LA1589 (*Solanum pimpinellifolium*) (Figure 1C). Seven first-generation transgenic (T_0_) individuals were obtained (Supplementary Figure 2). The disrupted target sites detected by PCR and Sanger sequencing suggested that two plants were biallelic for *m3_+1*bp*_*/*m9_–5*bp*_* (T_0_–1) or *m3_+1*bp*_*/*m6_–2*bp*_* (T_0_–15), two plants were homozygous for *m6_–2*bp*_* (T_0_–9) or *m16_–10*bp*_* (T_0_–11), and other two plants were heterozygous for *m3_+1*bp*_* (T_0_–12) or chimeric for at least three alleles (T_0_–10) (Supplementary Figure 2 and Supplementary Table 1).

**FIGURE 2:**
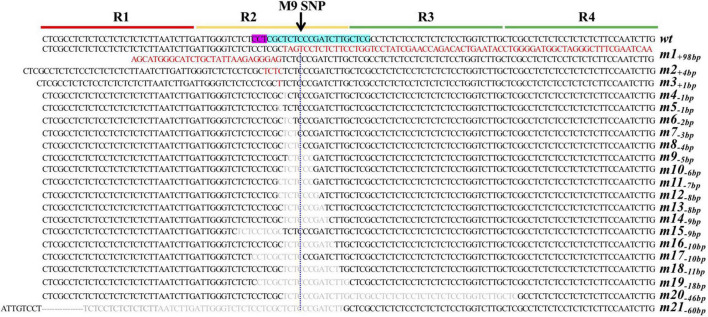
CRISPR/Cas9-induced mutations in LA1589. PAM and gRNA are highlighted in pink and cyan, respectively. The four tandem repeats in promoter were indicated by R1, R2, R3, and R4. The black arrow and blue dashed line indicate the position of the M9 SNP in LA1589 and all the 21 mutants, respectively. Inserted and deleted nucleotides were shown in red and gray, respectively. m, mutant allele. The mutant alleles were numbered 1 through 21 according to the length of the alleles. For the subscripts of the mutant alleles, (-) indicates deletions followed by the number of bp deleted, (+) indicates insertions followed by the number of bp inserted.

To enrich for *SlKLUH* promoter mutant alleles covering a range of fruit weight variations, the CRISPR/Cas9-driven mutagenesis approach was utilized (Rodriguez-Leal et al., 2017). A sensitized population of 719 F_1_ plants was generated by the backcrossing of T_0_ lines with LA1589 and genotyped by PCR and Restriction Enzyme (PCR/RE) analysis (Supplementary Figure 3). F_1_ progenies from T_0_–9 crossed to LA1589 resulted in the identification of three new alleles, namely, *m2_+4*bp*_*, *m14_–9 *bp*_*, and *m21_–60 *bp*_*, based on indels observed by gel electrophoresis mobility shift after PCR using primers flanking the target region (Figure 2; Supplementary Figure 3; Supplementary Table 2). To identify small indels that are different from *m6_–2*bp*_* or *wt*, we developed a screen scheme that exploits dCAPS analysis according to the sequences of *m6_–2*bp*_* and *wt* and the recognition sequences of the three REs (*Bsr*BI, *Hae*III, and *Aci*I). The remaining progenies were genotyped by dCAPS-*Bsr*BI, which can digest *m6_–2*bp*_* and *wt*. The new mutations that do not carry the restriction site for the digestion with *Bsr*BI were further analyzed by dCAPS-*Hae*III and/or dCAPS-*Aci*I, by which we identified one plant heterozygous for *m8_–4*bp*_* (Figure 2; Supplementary Figure 4; Supplementary Table 2). With this approach, we identified another 13 new alleles from the progenies of T_0_–10, T_0_–12, and T_0_–15 (Figure 2; Supplementary Figure 4; Supplementary Table 2).

Collectively, a total of 21 mutant alleles were created in the LA1589 background (Figure 2 and Supplementary Table 2), including three insertion alleles (1, 4, and 98 bp) and deletions of various sizes (from 1 to 60 bp) (Figure 2). Cas9 often cleaves double-strand DNA at a position of 3-bp upstream of the protospacer adjacent motif (PAM) sequence, and most mutations occurred at the 4th base from the PAM site (Jinek et al., 2012). However, we found that eight alleles (*m5_–1*bp*_*, *m9_–5*bp*_*, *m11_–7*bp*_*, *m12_–8*bp*_*, *m15_–9*bp*_*, *m17_–10*bp*_*, *m19_–18*bp*_*, and *m21_–60*bp*_*) did not occur right upstream of the predicted double-strand break (DSB) position. These results indicated the high efficiency of the CRISPR/Cas9-driven mutagenesis screen approach creating a collection of novel mutant alleles, including unexpected mutations, in tomatoes. F_1_ plants carrying novel alleles that were Cas9 positive were backcrossed to LA1589 to segregate away the Cas9 transgene to avoid further edits and minimize off-target effects. The Cas9-free mutants homozygous for these mutant alleles were obtained in F_2_ and F_3_ generations and used for further analysis.

### Evaluation of Novel *Cis*-Regulatory Alleles for Fruit Weight Variation

**FIGURE 3:**
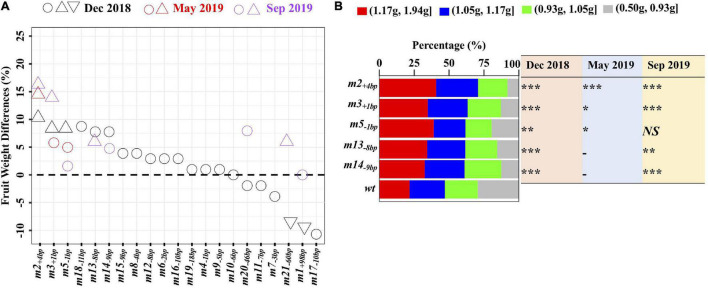
The effects of mutant alleles on fruit weight in three experimental evaluations. **(A)** Fruit weight differences from LA1589. Each data point represents the fruit weight differences of the mutant (in percentage) to the LA1589. Up and down triangle indicates significantly greater or lower than LA1589 (two-tailed Student’s *t*-test, *P* < 0.05), respectively, and the circle represents no significant difference of the mutant relative to LA1589; **(B)** Percentage distribution of the fruit weight according to fruit weight quartile. Fruits were grouped by their weight into four categories based on the quartiles of fruit weight in all genotypes in each replicate. Each quartile is indicated by a different color, and the fruit range within each quartile is shown in the upper part. A quantitative plot was generated using the data from December 2018. Asterisks denote significant difference (*P < 0.05; **P < 0.01; ***P < 0.001) of the proportion of small fruit (gray bar) between mutant and LA1589 as determined by chi-squared test. NS, non-significant difference; -, not evaluated.

The plants homozygous for the 21 alleles were grown in December 2018 with at least three plants per genotype (Supplementary Table 3). They displayed a continuum of fruit weight variation between 0.92 and 1.14 g (Figure 3A and Supplementary Table 3). Compared to LA1589, plants homozygous for nine alleles (*m2_+4*bp*_*, *m3_+1*bp*_*, *m5_–1*bp*_*, *m18_–11*bp*_*, *m13_–8*bp*_*, *m14_–9*bp*_*, *m21_–60*bp*_*, *m1_+98*bp*_*, and *m17_–10*bp*_*) showed more than 5% variation in fruit weight (Figure 3A). Notably, fruit weight of *m2_+4*bp*_*, *m3_+1*bp*_*, *m5_–1*bp*_*, *m18_–11*bp*_*, *m13_–8*bp*_*, and *m14_–9*bp*_* homozygotes was greater by 10.68, 8.74, 8.74, 8.74, 7.77, and 7.77% compared to LA1589, respectively. In contrast, fruit weight decreased by 8.74, 9.71, and 10.68% for *m21_–60*bp*_*, *m1_+98*bp*_*, and *m17_–10*bp*_*, respectively (Figure 3A and Supplementary Table 3).

We then selected eight mutant alleles for further analysis, including five larger-fruited alleles (*m2_+4*bp*_*, *m3_+1*bp*_*, *m5_–1*bp*_*, *m13_–8*bp*_*, and *m14_–9*bp*_*) and three smaller-fruited alleles with large insertion/deletions (*m1_+98*bp*_*, *m20_–46*bp*_*, and *m21_–60*bp*_*). Notably, statistically significant increases in average fruit weight were observed in plants homozygous for *m2_+4*bp*_* and *m3_+1*bp*_* (Figure 3A and Supplementary Table 3). The increased fruit weight of the homozygotes *m5_–1*bp*_*, *m13_–8*bp*_*, and *m14_–9*bp*_* was still observed and showed 5% or more variation in fruit weight compared to LA1589 in May 2019 and/or September 2019 (Figure 3A and Supplementary Table 3). Collectively, *m2_+4*bp*_*, *m3_+1*bp*_*, *m5_–1*bp*_*, *m13_–8*bp*_*, and *m14_–9*bp*_* homozygous mutants had 14.07, 9.60, 5.09, 6.26, and 7.06% greater fruit weight compared to the LA1589 (Figure 3A and Supplementary Table 3). However, the smaller-fruited alleles with large insertions/deletions, *m1_+98*bp*_*, *m20_–46*bp*_*, and *m21_–60*bp*_*, had inconsistent effects on fruit weight between December 2018 and September 2019 (Figure 3A and Supplementary Table 3), potentially due to environmental effects. In the following experiments, we focused only on the larger fruit mutant alleles (*m2_+4*bp*_*, *m3_+1*bp*_*, *m5_–1*bp*_*, *m13_–8*bp*_*, and *m14_–9*bp*_*) as they showed consistent effects on fruit weight across all experimental evaluations.

To better describe the increased fruit weight in the mutants homozygous for *m2_+4*bp*_*, *m3_+1*bp*_*, *m5_–1*bp*_*, *m13_–8*bp*_*, and *m14_–9*bp*_*, we performed a fruit weight distribution analysis. Fruits were grouped into four categories based on the quartiles of fruit weight in all genotypes in each experimental evaluation. The mutant alleles displayed a range of quantitative effects on the distribution of fruit weight in each experimental evaluation (Supplementary Figure 5). Importantly, the five large-fruited alleles (*m2_+4*bp*_*, *m3_+1*bp*_*, *m5_–1*bp*_*, *m13_–8*bp*_*, and m14*_–9*bp*_*) showed a significant decrease in the proportion of the small fruits (gray bar) across all or the majority of the experimental evaluations (Figure 3B and Supplementary Figure 5). The results indicated that the decreased proportion of the small fruits is responsible for the increased fruit weight in the five mutants.

### The Effects of the Five Mutant Alleles on Fruit Weight Based on Fruit Position on Inflorescence

Previous studies in the domesticated tomato demonstrated that fruits in the same inflorescence generally differ in size from the larger ones at the proximal position (Beadle, 1937; Bangerth and Ho, 1984) and similar results were also observed in LA1589 (Supplementary Figure 6). Fruit weight showed a decreasing trend from the 1st (proximal) to 8th (distal) fruit in the inflorescence, and the decrease rate of fruit weight was 29.1% in December 2018, 35.0% in May 2019, and 13.8% in September 2019 (Supplementary Figure 6).

**FIGURE 4:**
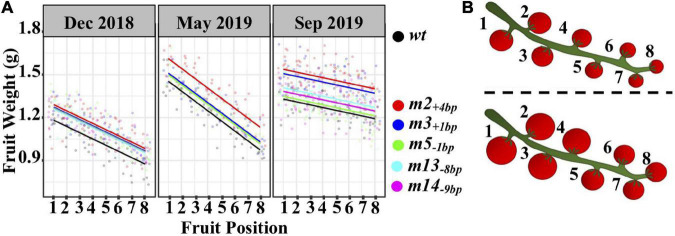
The effects of genotype and fruit position on fruit weight. **(A)** Linear regression between fruit weight and fruit position. Because the interaction of genotype and fruit position had no significant effect, the slopes of the regression lines are equal; in other words, the regression lines are parallel to each other in each replicate. Each data point is the mean fruit weight of each fruit position in one plant; **(B)** Schematic diagram shows the increased fruit weight along inflorescence in the mutants (lower panel) compared to LA1589 (upper panel).

Given that the five mutant alleles can increase fruit weight and decrease the proportion of small fruits, we hypothesized that this could be achieved by reducing the decreasing trend along with the position of an individual inflorescence or increasing the weight of all fruits on each inflorescence. To explore this, two-way ANOVA was performed using the weight and position along the inflorescence. As expected, the fruit weight was significantly affected by genotype and fruit position (*P* < 0.05) (Table 1). However, the interaction of genotype and fruit position had no significant effect (*P* > 0.05) in all experimental evaluations (Table 1 and Figure 4), indicating that the five mutant alleles had no significant effect on the rate of fruit weight decrease along inflorescence. Therefore, the five mutant alleles can increase the weight of all fruits along inflorescence without affecting the decreasing rate.

**TABLE 1:** Results of two-way ANOVA and Tukey’s honestly significant difference (HSD) test examining the influence of genotype and fruit position along inflorescence on fruit weight in each replication.

Two-way ANOVA	December 2018				May 2019				September 2019			
	Df	Sum sq	*F* value	*P*	Df	Sum sq	*F* value	*P*	Df	Sum sq	*F* value	*P*
Genotype	5	0.43	11.37	0.00*	3	0.64	32.21	0.00*	5	2.61	53.35	0.00*
Fruit position	7	3.00	56.89	0.00*	7	4.56	97.96	0.00*	7	1.10	15.98	0.00*
Genotype*Fruit position	35	0.15	0.57	0.98	21	0.05	0.37	0.99	35	0.11	0.31	1.00

*Significant difference (P < 0.05). Different color indicates different experimental evaluations.

### Non-linear Relationships Between Allele Types, Expression Levels, and Phenotypic Changes

The effects of promoter mutations on the phenotypic changes are often unpredictable and unexpected due to the complexity of transcriptional controls (Rodriguez-Leal et al., 2017). In this study, eight alleles (*m1_+98*bp*_*, *m2_+4*bp*_*, *m3_+1*bp*_*, *m4_–1*bp*_*, *m5_–1*bp*_*, *m6_–2*bp*_*, *m7_–3*bp*_*, and *m15_–9*bp*_*) had the unaltered *wt* M9 SNP (Figure 2), among which *m2_+4*bp*_*, *m3_+1*bp*_*, and *m5_–1*bp*_* showed an increase in fruit weight, while other five showed no or inconsistent effects on fruit weight in this study (Figure 3 and Supplementary Table 3). These results suggested that the M9 SNP had no or minor effects on tomato fruit weight, which was supported by the finding of the *SlKLUH* gene duplication underlying *fw3.2*(Alonge et al., 2020). In addition, although many alleles shared overlapping deletions, they had different phenotypic effects. For example, *m13*_–8*bp*_ and *m14_–9*bp*_* showed consistent effects on increasing fruit weight, while *m20_–46*bp*_* and *m21_–60*bp*_* had opposite effects on fruit weight between December 2018 and September 2019 (Figure 3A and Supplementary Table 3).

**FIGURE 5:**
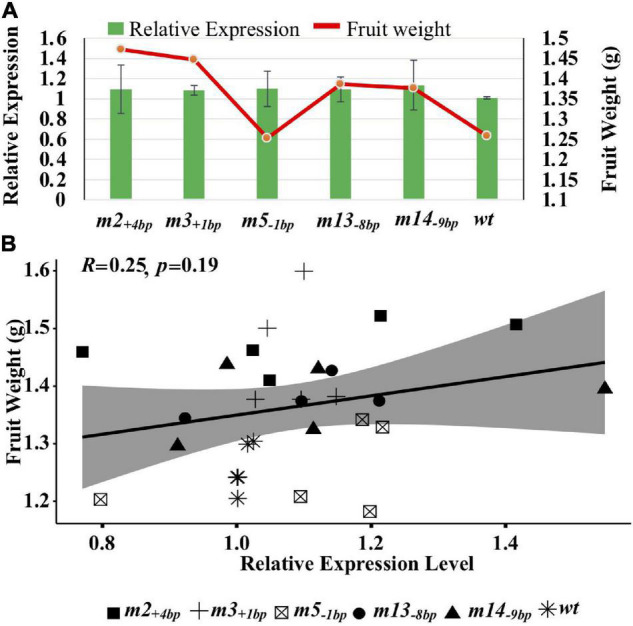
Non-linear relationships between transcriptional change for *SlKLUH* and fruit weight variation. **(A)** *SlKLUH* expression and fruit weight analyses of the *m2_+4*bp*_*, *m3_+1*bp*_*, *m5_–1*bp*_*, *m13_–8*bp*_*, and *m14_–9*bp*_* homozygotes; **(B)** The correlation between *SlKLUH* expression and fruit weight. Each point represents one plant per genotype. Two experimental evaluations were taken, each with four to five plants per genotype. The *m13_–8*bp*_* and *m14_–9*bp*_* homozygotes were only included in one experimental evaluation.

Given that the lower expression of *SlKLUH* results in smaller fruits (Chakrabarti et al., 2013; Alonge et al., 2020), we tested whether the expression levels of *SlKLUH* were upregulated in the mutants homozygous for the five larger fruit mutant alleles. We evaluated the *SlKLUH* expression in young flower buds at 9–13 dpi (Supplementary Figure 7). Although increased fruit weight was observed for the five large-fruited mutant alleles compared to LA1589, the *SlKLUH* expression levels from them are comparable to LA1589 (Figure 5A). Remarkably, there was a low correlation between the *SlKLUH* expression levels and fruit weights (Figure 5B). Similar findings were also observed for the tomato *lc* allele that is caused by two SNPs in a 15-bp repressor element downstream of tomato *WUSCHEL* (*SlWUS*) (van der Knaap et al., 2014; Rodriguez-Leal et al., 2017). Subtle differences in *SlWUS* expression level were not captured by RT-PCR, resulting in larger fruit size (Muños et al., 2011; van der Knaap et al., 2014; Rodriguez-Leal et al., 2017). These results support that allele types and transcriptional changes are poor predictors of phenotypic changes as previously noted (Rodriguez-Leal et al., 2017; Hendelman et al., 2021).

## Discussion

Expanding genetic diversity is of great importance for fine-tuning quantitative traits. However, the reduced genetic variation in modern crops limits the resources that breeders have available to cause subtle changes in agronomic traits (Birchler, 2017; Xing et al., 2020). CRISPR/Cas-mediated *cis*-engineering holds great promise to fine-tune quantitative traits that are highly valued in crop improvement such as yield and produce size (Rodriguez-Leal et al., 2017; Pandiarajan and Grover, 2018; Wolter and Puchta, 2018; Li Q. et al., 2020).

Mutations in the promoter often result in unexpected transcriptional and phenotypic changes due to the complexity of transcriptional control (Rodriguez-Leal et al., 2017; Shrestha et al., 2018; Cui et al., 2020; Huang et al., 2020). This is especially true for editing promoters with several gRNAs or unknown CREs. Contrary to this, mutating CREs with known functions can generate predictable quantitative variation or traits. One remarkable example is the disruption of the CArG element, a repressor motif downstream of *SlWUS*, leading to larger fruits with more locules (Rodriguez-Leal et al., 2017; Li et al., 2018). Two recent studies in rice reported that bacterial blight-resistant plants were created through CRISPR/Cas editing of the transcription-activator-like effector (TALe)-binding element (EBE) in the promoter of *SUGARS WILL EVENTUALLY BE EXPORTED TRANSPORTERS* (*SWEET*) genes (Oliva et al., 2019; Li C. et al., 2020). In another example in rice, the modification of the GT-1 element that is responsible for salt induction of *OsRAV2* confers adaptive salt responses (Duan et al., 2016). These studies highlight the great value for *de novo* discovery and characterization of CREs for boosting CRISPR/Cas-mediated *cis*-engineering in crop improvement.

The identification of conserved motifs by comparing promoter sequences of orthologous genes from different plants is one of the effective ways of *de novo* CRE discovery (Li Q. et al., 2020). Initially, no well-known or previously described CREs were identified in the target site using PLACE and PlantCARE. However, three conserved motifs that correspond to the four tandem repeats in the *SlKLUH* promoter were identified by the comparative analysis of the orthologous *KLUH* promoters. These data, together with the results of the ATAC-seq, directed us to engineer the specific CRE including the M9 SNP using CRISPR/Cas9. While knockout or constitutive knockdown of *SlKLUH* leads to growth defects and infertile phenotypes (Chakrabarti et al., 2013; Alonge et al., 2020), all the novel promoter alleles generated had normal growth and fertility in our study, supporting the notion that the modifications of *cis*-regulatory regions can benefit crop improvement or breeding with no or less deleterious effects (Swinnen et al., 2016; Rodriguez-Leal et al., 2017; Li Q. et al., 2020). Overall, the deletion alleles had no or weak positive effects on fruit weight, whereas the alleles with insertions except for *m1_+98*bp*_* significantly increased fruit weight. Importantly, consistent and reproducible results were observed for the five mutant alleles showing significant changes in fruit weight, indicating that the deletions and insertions may have generated novel CREs in the *SlKLUH* promoter, resulting in larger fruit without a detrimental impact, especially for the 1- and 4-bp insertions. However, the mechanistic aspects of increased fruit weight are not known. Therefore, *m2_+4*bp*_* and *m3_+1*bp*_* show great potential for breeding by introducing them into elite tomato cultivars using precision genome editing. In addition, the conserved motifs were also identified in the promoter of *KLUH*s in potato, pepper, soybean, rice, wheat, maize, and sweet cherry (Figure 1), suggesting that our approach could be applicable to engineer fruit or seed size variation with *KLUH*s in these crops.

Promoter editing has revealed complex relationships between allele types, transcriptional changes, and phenotypic changes which remain to be fully elucidated (Rodriguez-Leal et al., 2017). However, they can have positive effects on agronomically important traits. In this study, we observed positive changes in fruit weight in the five large-fruited mutants, especially mutants homozygous for *m2_+4*bp*_* and *m3_+1*bp*_*; however, no simple linear relationship between expression level and fruit weight changes was observed (Figures 3, 5). This may be due to the complexity of transcriptional control and the pleiotropic regulation of genes by differing elements within the promoter to control the additional aspects of gene expression, such as spatial-temporal specificity, and has been observed in other studies editing multiple CREs (Rodriguez-Leal et al., 2017; Hendelman et al., 2021; Liu et al., 2021). It is possible that larger fruits are due to changes in *SlKLUH* expression at developmental stages not sampled or at levels not detectible by qRT-PCR, such as changes in cell types expressing *SlKLUH*. Therefore, using CRISPR/Cas-mediated promoter engineering to screen for desirable traits at the phenotypic level may be more practical for crop improvement than detecting transcriptional changes. Moreover, the role of the putative CRE harboring the M9 SNP in fruit weight regulation needs to be further investigated.

In summary, this study identified a putative conserved CRE by combining homology-based prediction, ATAC-seq, and CRISPR/Cas9, which is applicable to diverse genes and crops. Five alleles, namely, *m2_+4*bp*_*, *m3_+1*bp*_*, *m5_–1*bp*_*, *m13_–8*bp*_*, and *m14_–9*bp*_*, were created by editing the CRE that showed increased fruit weight and potential for breeding. This study not only provides a way of identifying conserved CRE but also highlights the enormous potential for CRISPR/Cas-mediated *cis*-engineering of CYP78A members in yield improvement.

## Data Availability Statement

The original contributions presented in the study are included in the article/Supplementary Material, further inquiries can be directed to the corresponding authors.

## Author Contributions

QL and EK conceived the project. QL, QF, AS, BZ, and GR performed the research. QL analyzed the data and wrote the draft with revisions from QF, AS, BZ, GR, and EK. All authors contributed to the article and approved the submitted version.

## Conflict of Interest

The authors declare that the research was conducted in the absence of any commercial or financial relationships that could be construed as a potential conflict of interest.

## Publisher’s Note

All claims expressed in this article are solely those of the authors and do not necessarily represent those of their affiliated organizations, or those of the publisher, the editors and the reviewers. Any product that may be evaluated in this article, or claim that may be made by its manufacturer, is not guaranteed or endorsed by the publisher.

## Footnotes

1
http://cbi.hzau.edu.cn/cgi-bin/CRISPR

## Supplementary Material

Supplementary Figure 1 Sequence alignment of the four tandem repeats in *SlKLUH* promoter. The tandem repeats were identified by TANDEM REPEATS FINDER (https://tandem.bu.edu/trf/trf.basic.submit.html). In each pair of lines, the actual sequence is on the top and a consensus sequence for all the copies is on the bottom. The numbers at the beginning of the actual sequences indicate the positions relative to the translation start codon starting from the adenosine (+1). Red letters indicate mismatches. Y with green highlight indicates the M9 SNP (C-Wild type; T-Cultivated).

Supplementary Figure 2 PCR genotyping of T_0_ transgenic lines. Blue arrowhead indicated heteroduplex.

Supplementary Figure 3 Detection novel mutant alleles in the target site. Left panel, Sequences of the six mutant alleles with small indels (less than 5 bp) and recognition sequences of the three restriction enzymes (REs). Inserted and deleted nucleotide(s) were shown in red and gray, respectively. The yellow highlighted nucleotides indicate the mismatches to the wild-type (*wt*) allele. Right panel, Schematic of the PCR and RE assay. The short lines with different colors and dash types indicate the alleles that were shown in the left panel.

Supplementary Figure 4 PCR/RE analysis-based genotyping of F_1_ plants. The small indels that cannot be easily observed by gel electrophoresis mobility shift using PCR assays will be genotyped by dCAPS-*Bsr*BI, dCAPS-*Hae*III, and dCAPS-*Aci*I. The primers of PCR/RE assays were listed in Supplementary Table 4. The corresponding plant numbers of the alleles were shown in Supplementary Table 2.

Supplementary Figure 5 Fruit weight distribution analysis of mutants in LA1589 background in December 2018 **(A)**, May 2019 **(B)**, and September 2019 **(C)**. Data are presented as the percentage of fruits per fruit weight category. Asterisks denote significant difference (**P* < 0.05; ^**^*P* < 0.01; ^***^*P* < 0.001) of the proportion of small fruit (gray bar) between mutants and LA1589 as determined by chi-squared test.

Supplementary Figure 6 Fruit weight analysis based on fruit position on inflorescence in LA1589. **(A)** Fruit weight decreases from proximal to the distal end. Fruit position 1–8 indicates the 1st (proximal) to 8th (distal) fruit on inflorescence as shown in panel **(B)**.

Supplementary Figure 7 Young flower buds at 9–13 days post initiation (dpi) indicated by red arrows are used for *SlKLUH* expression quantification.

## References

[B1] Alonge M. Wang X. Benoit M. Soyk S. Pereira L. Zhang L. (2020). Major impacts of widespread structural variation on gene expression and crop improvement in tomato. *Cell* 182 145–161.e23. 10.1016/j.cell.2020.05.021 32553272 PMC7354227

[B2] Bangerth F. Ho L. (1984). Fruit position and fruit set sequence in a truss as factors determining final size of tomato fruits. *Ann. Bot.* 53 315–320.

[B3] Beadle N. C. W. (1937). Studies in the growth and respiration of tomato fruits and their relationship to carbohydrate Content. *Aus. J. Exp. Biol. Med. Sci.* 15 173–190. 10.1002/jsfa.4044 20597097

[B4] Birchler J. A. (2017). Editing the phenotype: a revolution for quantitative genetics. *Cell* 171 269–270. 10.1016/j.cell.2017.09.024 28985558

[B5] Chakrabarti M. Zhang N. Sauvage C. Munos S. Blanca J. Canizares J. (2013). A cytochrome P450 regulates a domestication trait in cultivated tomato. *Proc. Natl. Acad. Sci. U.S.A.* 110 17125–17130. 10.1073/pnas.1307313110 24082112 PMC3801035

[B6] Chen K. Wang Y. Zhang R. Zhang H. Gao C. (2019). CRISPR/Cas genome editing and precision plant breeding in agriculture. *Annu. Rev. Plant Biol.* 70 667–697. 10.1146/annurev-arplant-050718-100049 30835493

[B7] Cui Y. Hu X. Liang G. Feng A. Wang F. Ruan S. (2020). Production of novel beneficial alleles of a rice yield-related QTL by CRISPR/Cas9. *Plant Biotechnol. J.* 18 1987–1989. 10.1111/pbi.13370 32115804 PMC7540660

[B8] Doebley J. F. Gaut B. S. Smith B. D. (2006). The molecular genetics of crop domestication. *Cell* 127 1309–1321. 10.1016/j.cell.2006.12.006 17190597

[B9] Duan Y. B. Li J. Qin R. Y. Xu R. F. Li H. Yang Y. C. (2016). Identification of a regulatory element responsible for salt induction of rice OsRAV2 through ex situ and in situ promoter analysis. *Plant Mol. Biol.* 90 49–62. 10.1007/s11103-015-0393-z 26482477

[B10] Gao C. (2018). The future of CRISPR technologies in agriculture. *Nat. Rev. Mol. Cell Biol.* 19 275–276. 10.1038/nrm.2018.2 29382940

[B11] Godfray H. C. J. Beddington J. R. Crute I. R. Haddad L. Lawrence D. Muir J. F. (2010). Food security: the challenge of feeding 9 billion people. *Science* 327 812–818. 10.1126/science.1185383 20110467

[B12] González-Aguilera K. L. Saad C. F. Chávez Montes R. A. Alves-Ferreira M. De Folter S. (2016). Selection of reference genes for quantitative real-time RT-PCR studies in tomato fruit of the genotype MT-Rg1. *Front. Plant Sci.* 7:1386. 10.3389/fpls.2016.01386 27679646 PMC5021083

[B13] Gupta S. Van Eck J. (2016). Modification of plant regeneration medium decreases the time for recovery of Solanum lycopersicum cultivar M82 stable transgenic lines. *Plant Cell Tissue Organ Cult. (PCTOC)* 127 417–423.

[B14] Hendelman A. Zebell S. Rodriguez-Leal D. Dukler N. Robitaille G. Wu X. (2021). Conserved pleiotropy of an ancient plant homeobox gene uncovered by cis-regulatory dissection. *Cell* 184 1724–1739.e16. 10.1016/j.cell.2021.02.001 33667348

[B15] Hickey L. T. Hafeez A. N. Robinson H. Jackson S. A. Leal-Bertioli S. C. Tester M. (2019). Breeding crops to feed 10 billion. *Nat. Biotechnol.* 37 744–754. 10.1038/s41587-019-0152-9 31209375

[B16] Holme I. B. Wendt T. Gil-Humanes J. Deleuran L. C. Starker C. G. Voytas D. F. (2017). Evaluation of the mature grain phytase candidate HvPAPhy_a gene in barley (*Hordeum vulgare* L.) using CRISPR/Cas9 and TALENs. *Plant Mol. Biol.* 95 111–121. 10.1007/s11103-017-0640-6 28755320

[B17] Hua K. Zhang J. Botella J. R. Ma C. Kong F. Liu B. (2019). Perspectives on the application of genome editing technologies in crop breeding. *Mol. Plant*. 12 1047–1059. 10.1016/j.molp.2019.06.009 31260812

[B18] Huang L. Li Q. Zhang C. Chu R. Gu Z. Tan H. (2020). Creating novel Wx alleles with fine-tuned amylose levels and improved grain quality in rice by promoter editing using CRISPR/Cas9 system. *Plant Biotechnol. J.* 18 2164–2166. 10.1111/pbi.13391 32339389 PMC7589223

[B19] Hummel A. W. Chauhan R. D. Cermak T. Mutka A. M. Vijayaraghavan A. Boyher A. (2018). Allele exchange at the EPSPS locus confers glyphosate tolerance in cassava. *Plant Biotechnol. J.* 16 1275–1282. 10.1111/pbi.12868 29223136 PMC5999311

[B20] Jia H. Orbovic V. Wang N. (2019). CRISPR-LbCas12a-mediated modification of citrus. *Plant Biotechnol. J.* 17 1928–1937. 10.1111/pbi.13109 30908830 PMC6737016

[B21] Jia H. Orbovic V. Jones J. B. Wang N. (2016). Modification of the PthA4 effector binding elements in Type I CsLOB1 promoter using Cas9/sgRNA to produce transgenic Duncan grapefruit alleviating XccDeltapthA4:dCsLOB1.3 infection. *Plant Biotechnol. J.* 14 1291–1301. 10.1111/pbi.12495 27071672 PMC11389130

[B22] Jinek M. Chylinski K. Fonfara I. Hauer M. Doudna J. Charpentier E. (2012). A programmable dual-RNA–guided DNA endonuclease in adaptive bacterial immunity. *Science* 337 816–821. 10.1126/science.1225829 22745249 PMC6286148

[B23] Korotkova A. M. Gerasimova S. V. Shumny V. K. Khlestkina E. K. (2017). Crop genes modified using the CRISPR/Cas system. *Russ. J. Genet. Appl. Res.* 7 822–832.

[B24] Korotkova A. Gerasimova S. Khlestkina E. (2019). Current achievements in modifying crop genes using CRISPR/Cas system. *Vavilov J. Genet. Breed*. 23 29–37.

[B25] Kumar J. Gupta D. S. Gupta S. Dubey S. Gupta P. Kumar S. (2017). Quantitative trait loci from identification to exploitation for crop improvement. *Plant Cell Rep.* 36 1187–1213. 10.1007/s00299-017-2127-y 28352970

[B26] Lei Y. Lu L. Liu H.-Y. Li S. Xing F. Chen L.-L. (2014). CRISPR-P: a web tool for synthetic single-guide RNA design of CRISPR-system in plants. *Mol. Plant* 7 1494–1496. 10.1093/mp/ssu044 24719468

[B27] Li C. Li W. Zhou Z. Chen H. Xie C. Lin Y. (2020). A new rice breeding method: CRISPR/Cas9 system editing of the Xa13 promoter to cultivate transgene-free bacterial blight-resistant rice. *Plant Biotechnol. J.* 18 313–315. 10.1111/pbi.13217 31344313 PMC6953186

[B28] Li Q. Chakrabarti M. Taitano N. K. Okazaki Y. Saito K. Al-Abdallat A. M. (2021). Differential expression of SlKLUH controlling fruit and seed weight is associated with changes in lipid metabolism and photosynthesis-related genes. *J. Exp. Bot.* 72 1225–1244. 10.1093/jxb/eraa518 33159787 PMC7904157

[B29] Li Q. Sapkota M. Van Der Knaap E. (2020). Perspectives of CRISPR/Cas-mediated cis-engineering in horticulture: unlocking the neglected potential for crop improvement. *Hortic. Res.* 7:36. 10.1038/s41438-020-0258-8 32194972 PMC7072075

[B30] Li T. Yang X. Yu Y. Si X. Zhai X. Zhang H. (2018). Domestication of wild tomato is accelerated by genome editing. *Nat. Biotechnol*. 36 1160–1163. 10.1038/nbt.4273 30272676

[B31] Li X. Xie Y. Zhu Q. Liu Y. G. (2017). Targeted genome editing in genes and cis-regulatory regions improves qualitative and quantitative traits in crops. *Mol. Plant* 10 1368–1370. 10.1016/j.molp.2017.10.009 29079543

[B32] Liu L. Gallagher J. Arevalo E. D. Chen R. Skopelitis T. Wu Q. (2021). Enhancing grain-yield-related traits by CRISPR–Cas9 promoter editing of maize CLE genes. *Nat. Plants* 7 287–294. 10.1038/s41477-021-00858-5 33619356

[B33] Long S. P. Marshall-Colon A. Zhu X.-G. (2015). Meeting the global food demand of the future by engineering crop photosynthesis and yield potential. *Cell* 161 56–66. 10.1016/j.cell.2015.03.019 25815985

[B34] Lu Z. Marand A. P. Ricci W. A. Ethridge C. L. Zhang X. Schmitz R. J. (2019). The prevalence, evolution and chromatin signatures of plant regulatory elements. *Nat. Plants* 5 1250–1259. 10.1038/s41477-019-0548-z 31740772

[B35] Ma M. Wang Q. Li Z. Cheng H. Li Z. Liu X. (2015a). Expression of Ta CYP 78A3, a gene encoding cytochrome P450 CYP 78A3 protein in wheat (*Triticum aestivum* L.), affects seed size. *Plant J.* 83 312–325. 10.1111/tpj.12896 26043144

[B36] Ma M. Zhao H. Li Z. Hu S. Song W. Liu X. (2015b). TaCYP78A5 regulates seed size in wheat (*Triticum aestivum*). *J. Exp. Bot.* 67 1397–1410. 26712825 10.1093/jxb/erv542

[B37] Ma X. Chen L. Zhu Q. Chen Y. Liu Y. G. (2015c). Rapid decoding of sequence-specific nuclease-induced heterozygous and biallelic mutations by direct sequencing of PCR products. *Mol. Plant* 8 1285–1287. 10.1016/j.molp.2015.02.012 25747846

[B38] Maeda S. Dubouzet J. G. Kondou Y. Jikumaru Y. Seo S. Oda K. (2019). The rice CYP78A gene BSR2 confers resistance to *Rhizoctonia solani* and affects seed size and growth in Arabidopsis and rice. *Sci. Rep.* 9:587. 10.1038/s41598-018-37365-1 30679785 PMC6345848

[B39] Mao Y. Botella J. R. Liu Y. Zhu J.-K. (2019). Gene editing in plants: progress and challenges. *Natl. Sci. Rev.* 6 421–437. 10.1093/nsr/nwz005 34691892 PMC8291443

[B40] Morineau C. Bellec Y. Tellier F. Gissot L. Kelemen Z. Nogué F. (2017). Selective gene dosage by CRISPR-Cas9 genome editing in hexaploid *Camelina sativa*. *Plant Biotechnol. J.* 15 729–739. 10.1111/pbi.12671 27885771 PMC5425392

[B41] Muños S. Ranc N. Botton E. Bérard A. Rolland S. Duffé P. (2011). Increase in tomato locule number is controlled by two single-nucleotide polymorphisms located near WUSCHEL. *Plant Physiol.* 156 2244–2254. 10.1104/pp.111.173997 21673133 PMC3149950

[B42] Nagasawa N. Hibara K. I. Heppard E. P. Vander Velden K. A. Luck S. Beatty M. (2013). GIANT EMBRYO encodes CYP78A13, required for proper size balance between embryo and endosperm in rice. *Plant J.* 75 592–605. 10.1111/tpj.12223 23621326

[B43] Oliva R. Ji C. Atienza-Grande G. Huguet-Tapia J. C. Perez-Quintero A. Li T. (2019). Broad-spectrum resistance to bacterial blight in rice using genome editing. *Nat. Biotechnol.* 37 1344–1350. 10.1038/s41587-019-0267-z 31659337 PMC6831514

[B44] Pandiarajan R. Grover A. (2018). In vivo promoter engineering in plants: are we ready? *Plant Sci.* 277 132–138. 10.1016/j.plantsci.2018.10.011 30466578

[B45] Peng A. Chen S. Lei T. Xu L. He Y. Wu L. (2017). Engineering canker-resistant plants through CRISPR/Cas9-targeted editing of the susceptibility geneCsLOB1promoter in citrus. *Plant Biotechnol. J.* 15 1509–1519. 10.1111/pbi.12733 28371200 PMC5698050

[B46] Qi X. Liu C. Song L. Li Y. Li M. (2017). PaCYP78A9, a cytochrome P450, regulates fruit size in sweet cherry (*Prunus avium* L.). *Front. Plant Sci.* 8:2076. 10.3389/fpls.2017.02076 29259616 PMC5723407

[B47] Ray D. K. Mueller N. D. West P. C. Foley J. A. (2013). Yield trends are insufficient to double global crop production by 2050. *PLoS One* 8:e66428. 10.1371/journal.pone.0066428 23840465 PMC3686737

[B48] Rodriguez-Leal D. Lemmon Z. H. Man J. Bartlett M. E. Lippman Z. B. (2017). Engineering quantitative trait variation for crop improvement by genome editing. *Cell* 171 470–480.e8. 10.1016/j.cell.2017.08.030 28919077

[B49] Shi J. Lai J. (2015). Patterns of genomic changes with crop domestication and breeding. *Curr. Opin. Plant Biol.* 24 47–53. 10.1016/j.pbi.2015.01.008 25656221

[B50] Shrestha A. Khan A. Dey N. (2018). cis-trans engineering: advances and perspectives on customized transcriptional regulation in plants. *Mol. Plant* 11 886–898. 10.1016/j.molp.2018.05.008 29859265

[B51] Sun X. Cahill J. Van Hautegem T. Feys K. Whipple C. Novák O. (2017). Altered expression of maize PLASTOCHRON1 enhances biomass and seed yield by extending cell division duration. *Nat. Commun.* 8:14752. 10.1038/ncomms14752 28300078 PMC5356070

[B52] Swinnen G. Goossens A. Pauwels L. (2016). Lessons from domestication: targeting cis-regulatory elements for crop improvement. *Trends Plant Sci.* 21 506–515. 26876195 10.1016/j.tplants.2016.01.014

[B53] Tian Y. Zhang M. Hu X. Wang L. Dai J. Xu Y. (2016). Over-expression of CYP78A98, a cytochrome P450 gene from Jatropha curcas L., increases seed size of transgenic tobacco. *Electron. J. Biotechnol.* 19 15–22.

[B54] Tilman D. Balzer C. Hill J. Befort B. L. (2011). Global food demand and the sustainable intensification of agriculture. *Proc. Natl. Acad. Sci. U.S.A.* 108 20260–20264. 10.1073/pnas.1116437108 22106295 PMC3250154

[B55] van der Knaap E. Chakrabarti M. Chu Y. H. Clevenger J. P. Illa-Berenguer E. Huang Z. (2014). What lies beyond the eye: the molecular mechanisms regulating tomato fruit weight and shape. *Front. Plant Sci.* 5:227. 10.3389/fpls.2014.00227 24904622 PMC4034497

[B56] Wang X. Li Y. Zhang H. Sun G. Zhang W. Qiu L. (2015). Evolution and association analysis of GmCYP78A10 gene with seed size/weight and pod number in soybean. *Mol. Biol. Rep.* 42 489–496. 10.1007/s11033-014-3792-3 25324172

[B57] Willmann M. R. (2018). Will Plants Yield to CRISPR? *CRISPR J.* 1 211–213. 10.1089/crispr.2018.29020.wil 31021256

[B58] Wolter F. Puchta H. (2018). “Application of CRISPR/Cas to understand Cis-and trans-regulatory elements in plants,” in *Plant Transcription Factors. Methods in Molecular Biology*, Vol. 1830 ed. Yamaguchi N. (New York, NY: Humana Press), 23–40. 10.1007/978-1-4939-8657-6_2 30043362

[B59] Wolter F. Schindele P. Puchta H. (2019). Plant breeding at the speed of light: the power of CRISPR/Cas to generate directed genetic diversity at multiple sites. *BMC Plant Biol.* 19:176. 10.1186/s12870-019-1775-1 31046670 PMC6498546

[B60] Wu S. Zhang B. Keyhaninejad N. Rodriguez G. R. Kim H. J. Chakrabarti M. (2018). A common genetic mechanism underlies morphological diversity in fruits and other plant organs. *Nat. Commun.* 9:4734. 10.1038/s41467-018-07216-8 30413711 PMC6226536

[B61] Xing S. Chen K. Zhu H. Zhang R. Zhang H. Li B. (2020). Fine-tuning sugar content in strawberry. *Genome Biol.* 21:230. 10.1186/s13059-020-02146-5 32883370 PMC7470447

[B62] Xu C. Liberatore K. L. Macalister C. A. Huang Z. Chu Y.-H. Jiang K. (2015). A cascade of arabinosyltransferases controls shoot meristem size in tomato. *Nat. Genet.* 47:784. 10.1038/ng.3309 26005869

[B63] Yan S. Chen N. Huang Z. Li D. Zhi J. Yu B. (2020). Anthocyanin Fruit encodes an R2R3-MYB transcription factor, SlAN2-like, activating the transcription of SlMYBATV to fine-tune anthocyanin content in tomato fruit. *New Phyto.* 225 2048–2063. 10.1111/nph.16272 31625612

[B64] Yang W. Gao M. Yin X. Liu J. Xu Y. Zeng L. (2013). Control of rice embryo development, shoot apical meristem maintenance, and grain yield by a novel cytochrome p450. *Mol. Plant* 6 1945–1960. 10.1093/mp/sst107 23775595

[B65] Zhang N. (2012). *Fine Mapping and Characterization of fw3. 2, One of the Major QTL Controlling Fruit Size in Tomato.* Columbus, OH: The Ohio State University.

[B66] Zhang Y. Malzahn A. A. Sretenovic S. Qi Y. (2019). The emerging and uncultivated potential of CRISPR technology in plant science. *Nat. Plants* 5 778–794. 10.1038/s41477-019-0461-5 31308503

[B67] Zhao B. Dai A. Wei H. Yang S. Wang B. Jiang N. (2016). ArabidopsisKLU homologue GmCYP78A72 regulates seed size in soybean. *Plant Mol. Biol.* 90 33–47. 10.1007/s11103-015-0392-0 26482479

[B68] Zhu B. Zhang W. Zhang T. Liu B. Jiang J. (2015). Genome-wide prediction and validation of intergenic enhancers in *Arabidopsis* using open chromatin signatures. *Plant Cell* 27 2415–2426. 10.1105/tpc.15.00537 26373455 PMC4815101

[B69] Zhu H. Li C. Gao C. (2020). Applications of CRISPR-Cas in agriculture and plant biotechnology. *Nat. Rev. Mol. Cell Biol*. 21 661–677. 32973356 10.1038/s41580-020-00288-9

[B70] Zsogon A. Cermak T. Naves E. R. Notini M. M. Edel K. H. Weinl S. (2018). De novo domestication of wild tomato using genome editing. *Nat. Biotechnol*. 36 1211–1216. 10.1038/nbt.4272 30272678

[B71] Zuo J. Li J. (2014). Molecular genetic dissection of quantitative trait loci regulating rice grain size. *Annu. Rev. Genet.* 48 99–118. 10.1146/annurev-genet-120213-092138 25149369

