# Supplementary figures and images for: Increasing Fruit Weight by Editing a Cis-Regulatory Element in Tomato KLUH Promoter Using CRISPR/Cas9
Source: Front Plant Sci. 2022 Apr 11;13:879642. doi: 10.3389/fpls.2022.879642 (PMC9037380; doi:10.3389/fpls.2022.879642)

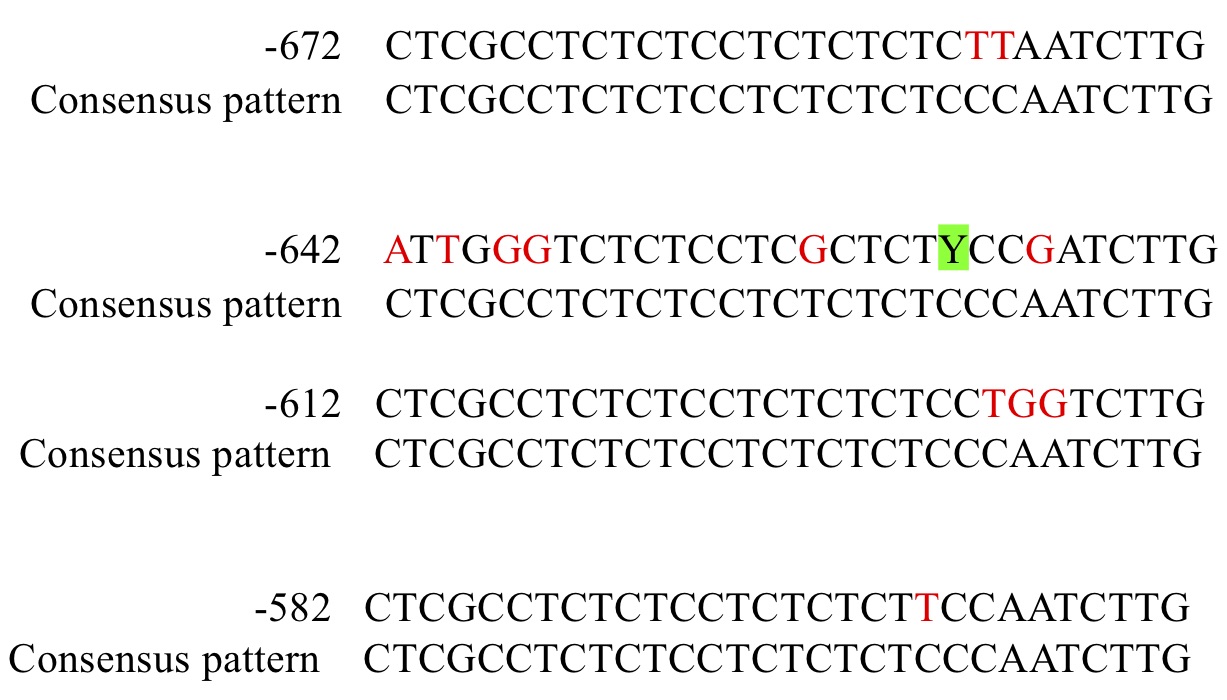

Supplement: Supplementary Figure 1 — Sequence alignment of the four tandem repeats in SlKLUH promoter. The tandem repeats were identified by TANDEM REPEATS FINDER (https://tandem.bu.edu/trf/trf.basic.submit.html). In each pair of lines, the actual sequence is on the top and a consensus sequence for all the copies is on the bottom. The numbers at the beginning of the actual sequences indicate the positions relative to the translation start codon starting from the adenosine (+1). Red letters indicate mismatches. Y with green highlight indicates the M9 SNP (C-Wild type; T-Cultivated). [file Image_1.JPEG]

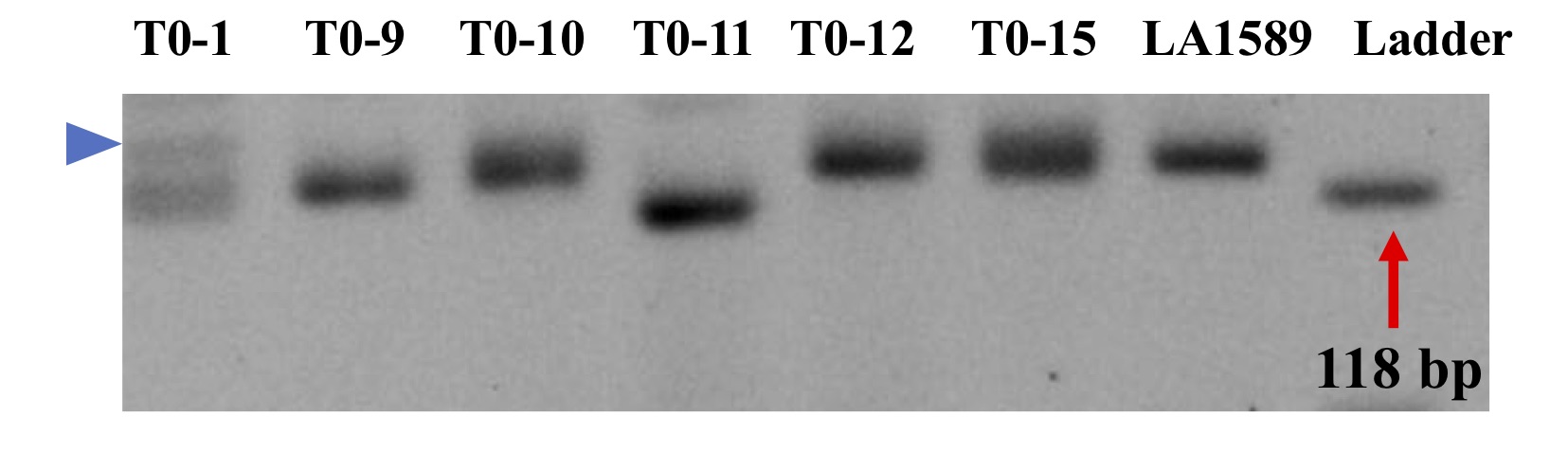

Supplement: Supplementary Figure 2 — PCR genotyping of T0 transgenic lines. Blue arrowhead indicated heteroduplex. [file Image_2.JPEG]

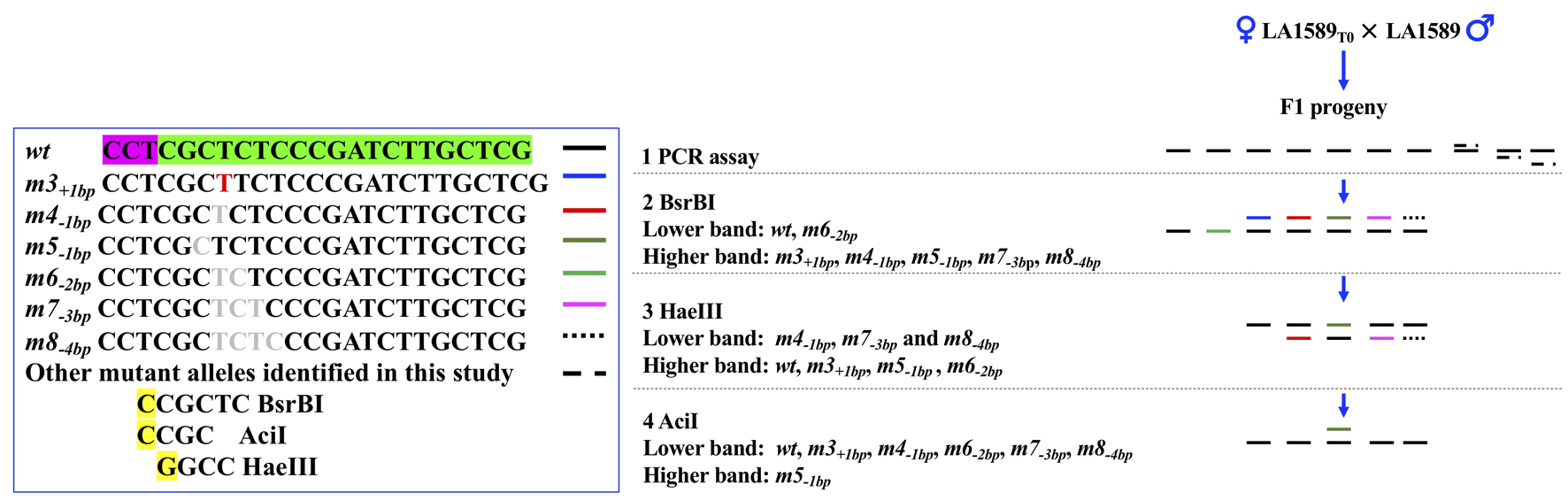

Supplement: Supplementary Figure 3 — Detection novel mutant alleles in the target site. Left panel, Sequences of the six mutant alleles with small indels (less than 5 bp) and recognition sequences of the three restriction enzymes (REs). Inserted and deleted nucleotide(s) were shown in red and gray, respectively. The yellow highlighted nucleotides indicate the mismatches to the wild-type (wt) allele. Right panel, Schematic of the PCR and RE assay. The short lines with different colors and dash types indicate the alleles that were shown in the left panel. [file Image_3.JPEG]

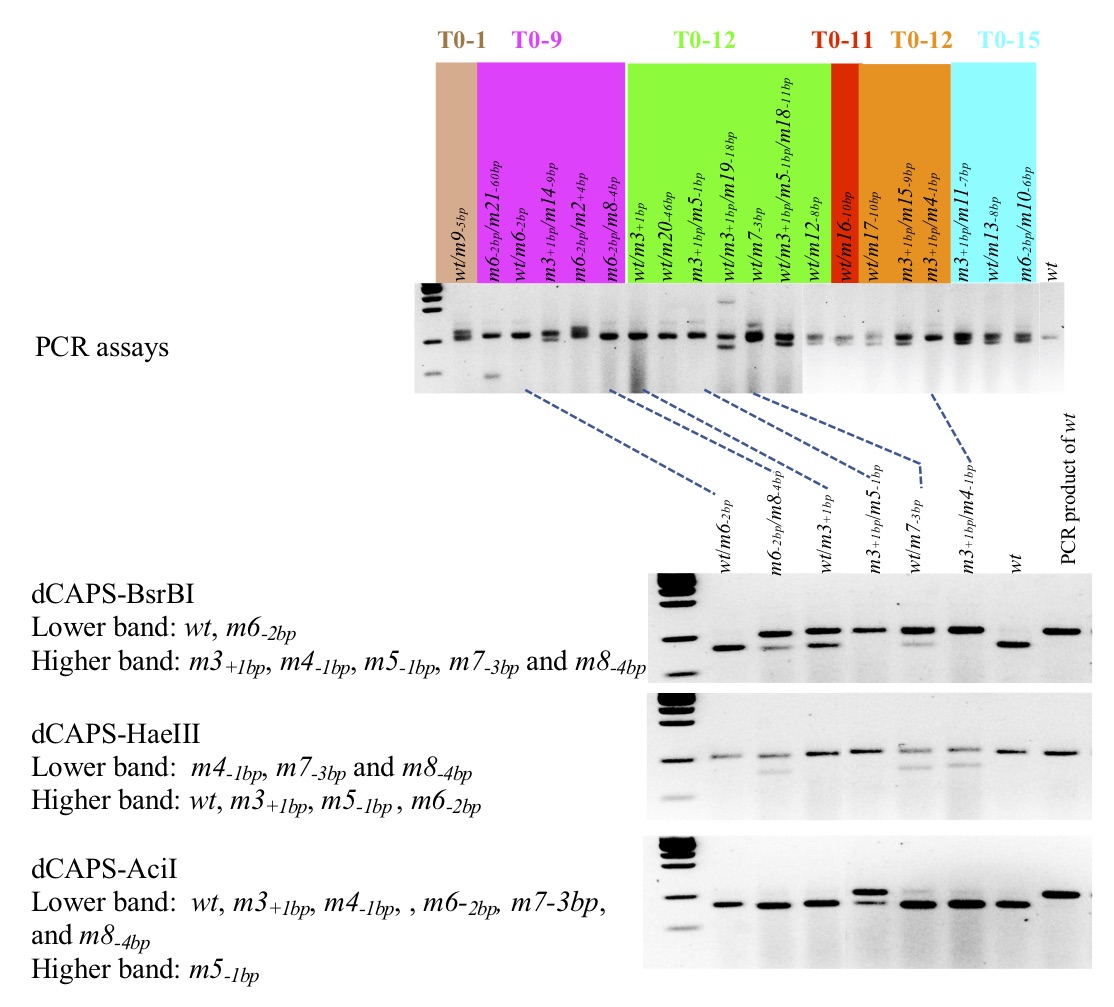

Supplement: Supplementary Figure 4 — PCR/RE analysis-based genotyping of F1 plants. The small indels that cannot be easily observed by gel electrophoresis mobility shift using PCR assays will be genotyped by dCAPS-BsrBI, dCAPS-HaeIII, and dCAPS-AciI. The primers of PCR/RE assays were listed in Supplementary Table 4. The corresponding plant numbers of the alleles were shown in Supplementary Table 2. [file Image_4.JPEG]

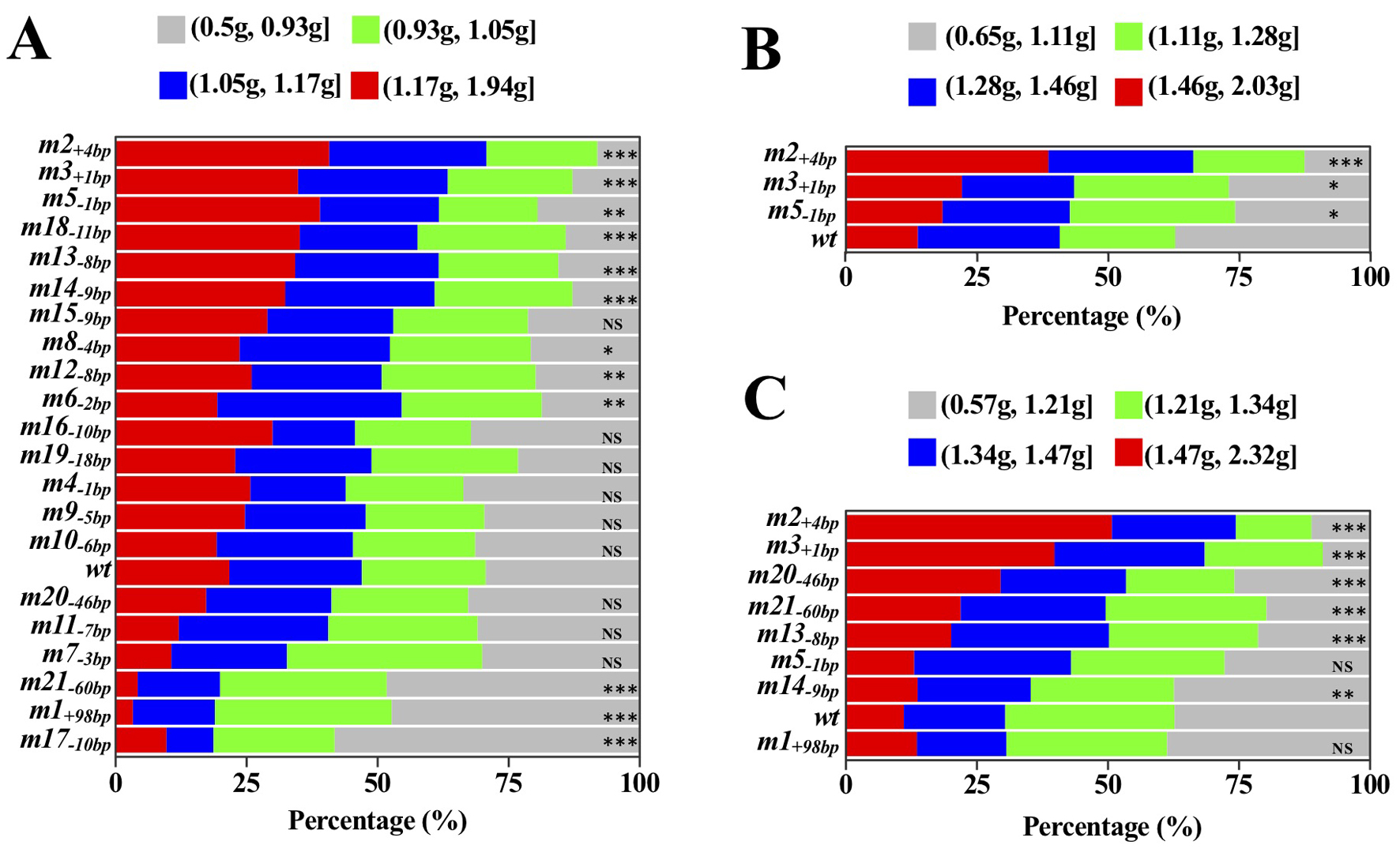

Supplement: Supplementary Figure 5 — Fruit weight distribution analysis of mutants in LA1589 background in December 2018 (A), May 2019 (B), and September 2019 (C). Data are presented as the percentage of fruits per fruit weight category. Asterisks denote significant difference (*P < 0.05; **P < 0.01; ***P < 0.001) of the proportion of small fruit (gray bar) between mutants and LA1589 as determined by chi-squared test. [file Image_5.JPEG]

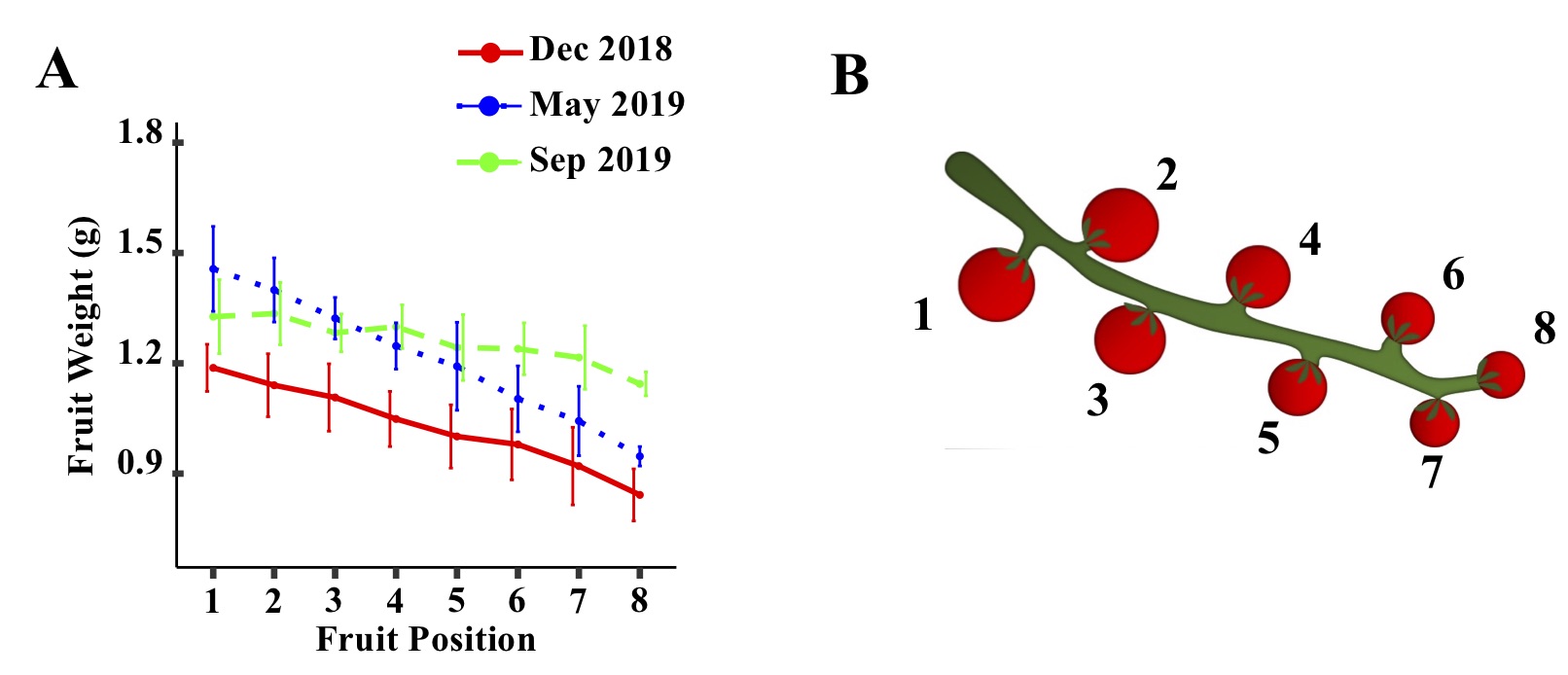

Supplement: Supplementary Figure 6 — Fruit weight analysis based on fruit position on inflorescence in LA1589. (A) Fruit weight decreases from proximal to the distal end. Fruit position 1–8 indicates the 1st (proximal) to 8th (distal) fruit on inflorescence as shown in panel (B). [file Image_6.JPEG]

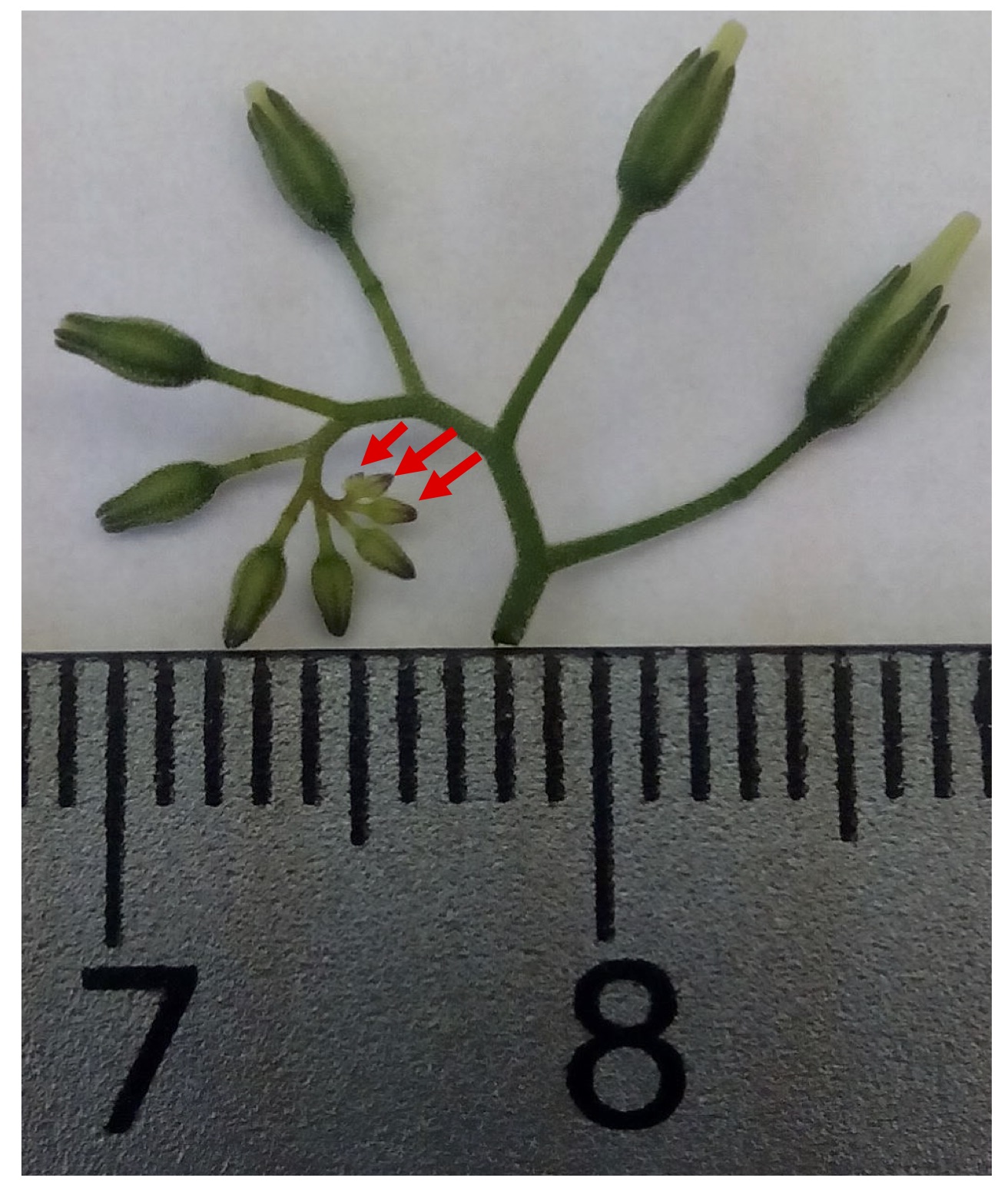

Supplement: Supplementary Figure 7 — Young flower buds at 9–13 days post initiation (dpi) indicated by red arrows are used for SlKLUH expression quantification. [file Image_7.JPEG]
